# Editorial: The psychological outcomes for leadership and employees in the education sector

**DOI:** 10.3389/fpsyg.2023.1325130

**Published:** 2023-11-29

**Authors:** Shumaila Naz

**Affiliations:** Lahore Business School, The University of Lahore, Lahore, Pakistan

**Keywords:** law, higher education institute (HEIs), teachers' self-efficacy, leadership style & leader-member exchange, turnover

The Research Topic aims to analyze leadership studies and their profound impact on organizational policy, decision-making, and employee behavior. Effective leadership shapes an organization's success by determining its direction, sustainability, and competitive advantages. However, the gap remains in understanding how a leader's actions affect followers. The exploration of these dynamics is crucial for obtaining practical implications, particularly in the field of education. Among the themes explored in this topic are positive and negative leadership effects, organizational behavior, and employee wellbeing in the education sector. Besides quantitative and qualitative studies, the topic encourages research that utilizes multiple sources of data, incorporates multiple levels of analysis, and employs multiple methods.

The studies in this topic have explored the relationship between a leader's actions and their effects on the health and wellbeing of their followers: (1) The role of higher education institutes in indorsing law-abiding behavior in students (Dong and Zeb). (2) How various leadership styles increase or decrease academic staff's job satisfaction (Kasalak et al.). (3) Explore the mediating role of organizational commitment in the relationship between ethical leadership and employees' ethical work behavior (Guo et al.). (4) Study examines the moderating effect of leader-member exchange (LMX) for increasing job satisfaction in post COVID-19 Pandemic in Palestinian Universities (Horoub and Zargar). (5) How to inspire teachers' enthusiasm for educational reform from the perspective of organizational support (Hsieh et al.). (6) A discussion regarding understanding the mediating effect of college teaching self-efficacy (CTSE) on the relationship between faculty job stress and job satisfaction (Liu et al.). (7) If supervisory styles are key predictors of graduate students' innovation capability and performance (Yang et al.).

Another study explores the role of higher educational institutions in the development of pupils' law-abiding behavior (Dong and Zeb). It has shed light on factors such as age, gender, education, occupation, and location that influence law-abiding behavior. Apart from law-abiding factor, HEI plays a significant part in the economic and social advancement of individuals (Popova and Popovs, 2022). The universities play a significant role in fostering positive changes in a student's life, nurturing intellectual and cognitive growth. Nevertheless, without appropriate guidance, students may adopt deviant behaviors, non-law abidance, and embrace detrimental beliefs. The author has proposed to establish a climate of respect and harmony to prevent the deviant behavior on the part of students, such as violence, drug use, and addictive behavior etc.

With ever-increasing competition, higher education examines new approaches of leadership as universities face several challenges to compete in a globalized world and develop sustainable leadership. Adoption of appropriate leadership style bring positive outcomes such as, the employees' retention, organizational justice and organizational trust, organizational commitment, and academic staff performance (Jameel and Ahmad, 2020). According to the previous research (Kasalak et al.), Spiritual leadership is characterized by love, compassion, honesty, harmony, unity, and peace and is ranked at the top of the leadership styles list that impact employee job satisfaction unswervingly. In addition, Passive leadership has a negative effect on academic staff satisfaction, whereas transactional leadership has a strong but limited effect.

According to a recent survey conducted in the United States in 2018, employees are concerned about the declining integrity and honesty of their leaders. Leaders who are empowered by the quality of ethical leadership treat their employees fairly and respectfully (Huo et al., 2022). A study conducted by Guo et al., highlights the increasing unethical behavior of Chinese employees, resulting in the higher costs to organizations such as, early departures, misuse of official computers, and use of office telephones for unofficial calls. The organization and leaders should maintain an effective environment to boost the employees' workplace ethical behavior.

China has been experiencing challenges such as poor academic results and a lack of innovation predominantly due to two following reasons: First, the increasing number of post graduates and second, procrastination in completion of their degree program. Here, the supervisor's role is of great significance for their academic performance. Harmonious academic passion (HAP) is considered vital individual characteristic which stimulates and persuades graduates to engage themselves in research pursuits. According to Yang et al. the learners with increased level of HAP produce more clear research proposals, hence bring more creativity in their academic endeavors.

Leadership that empowers individuals instead of focusing on traditional power approaches is considered a positive and ethical technique that leads to positive outcomes for employees and the organization. Leaders can strengthen other workers by promoting their societal and cognitive facets (Horoub and Zargar). The author affirms the view that applying Leader-member exchange (LMX) empowered leadership to heighten the job satisfaction of the employee and boost interaction with the teachers in academic settings, which will impact their wellbeing.

The academic field has observed a constant influx of reforms that have increased teachers' workload in educational settings. In such circumstances, maintaining teachers' enthusiasm to accept the change is crucial from an organizational perspective. The research by Hsieh et al. has explored the relationship between organizational support (OS), job engagement (JE), and organizational citizenship behavior (OCB) in an educational context. Teachers' retention in colleges and universities has become a great challenge for higher education institutions. Although it is a global concern, however, China witnesses a decline of retention level due to research incapabilities and teaching load that in turn derive anxiety, cognitive fatigue and burnout (Yin et al., 2020; Liu et al.).

This Research Topic has various contributions particularly the leadership impact on organizational aspects and how it affects employees' psychological, social, and ethical wellbeing. The different studies have extended the existing literature by introducing new dynamics by embedding ethical values, innovative leadership, and maintaining teachers' enthusiasm amidst educational challenges.

## Author contributions

SN: Conceptualization, Writing—original draft.
